# Protective Effect of *Cornus mas* Fruits Extract on Serum Biomarkers in CCl_4_-Induced Hepatotoxicity in Male Rats

**DOI:** 10.5812/hepatmon.10330

**Published:** 2014-04-14

**Authors:** Seyed Moayed Alavian, Nafiseh Banihabib, Masoud Es. Haghi, Farid Panahi

**Affiliations:** 1 Middle East Liver Disease Center (MELD), Tehran, IR Iran; 2 Liver and Gastrointestinal Diseases Research Center, School of Medicine, Tabriz University of Medical Sciences, Tabriz, IR Iran; 3 Baqiyatallah Research Center for Gastroenterology and Liver Disease (BRCGL), Baqiyatallah University of Medical Sciences, Tehran, IR Iran; 4 Drug Applied Research Center, School of Medicine, Tabriz University of Medical Sciences, Tabriz, IR Iran; 5 Coordination Center, School of Medicine, Tabriz University of Medical Sciences, Tabriz, IR Iran

**Keywords:** Carbon Tetrachloride, Cornus mas, Hepatotoxicity, Lipid Peroxidation, Serum Biomarkers

## Abstract

**Background:**

Nowadays attention to use herbs such as cornelian cherry (*Cornus mas*) is increasing, which contains high levels of antioxidants and anthocyanins. *Cornus mas* fruits have been used for gastrointestinal and excretory disorders for many years in traditional medicine, also may improve liver and kidney functions, and have protective effects such as anti-allergic, antidiabetic, antibacterial, antimicrobial, antihistamine and antimalarial properties.

**Objectives:**

The aim of this study was to investigate protective effects of *Cornus mas* fruits extract on serum biomarkers in CCl_4_-induced hepatotoxicity in male rats.

**Materials and Methods:**

Hepatotoxicity was induced by administration of carbon tetrachloride (1 mL/kg i.p.) in 1:1 dilution with olive oil. To evaluate the effect of *Cornus mas* fruits extract on disease progression, serum marker enzymes, serum total protein and albumin and liver lipid peroxidation were determined in CCl_4_-induced hepatotoxicity.

**Results:**

Oral administration of *Cornus mas* fruits extract to rats for 14 days provided a significant (P < 0.05) hepatoprotection by decreasing elevated serum level of enzymes, total serum protein, albumin and liver lipid peroxidation content.

**Conclusions:**

*Cornus mas* fruit extract effect may be due to including some antioxidant components, which caused membrane stabilizing and normalization of fluctuated biochemical profiles induced by CCl_4_ exposure. Our results validated the traditional use of *Cornus mas* in the treatment of liver disorders.

## 1. Background

Hepatic injuries lead to attenuation of metabolic functions regulated by liver, and has remained one of the serious health problems (1) threatening the human society. The pathogenesis of liver fibrosis is not clear, but reactive oxygen species (ROS) have important roles in liver pathological changes (2). Unsaturated fatty acids of biological cell membranes (a sensitive part of cells against free radicals) may be affected by peroxidation reaction, which leads to a decrease in the fluidity and disruption of membrane function and integrity, and finally serious pathological changes (3). Oxygen free radicals and oxygen free-radical generating agents such as hydrogen peroxide (H_2_O_2_), superoxide anion (O_2_^•-^) and hydroxyl radical (^•^OH) are known as ROS. During the metabolic processes, free radicals are usually generated especially oxygen-derived radicals with potency of oxidizing and damaging biomolecules (4). Biotransformed of trichloromethyl-free radical (CCl_3_^•^ or CCl_3_OO^•^) from carbon tetrachloride (CCl_4_) by hepatic microsomal cytochrome P450, is a well-known model compound for producing chemical hepatic injury (5-9). Overproduction of trichloromethyl-free radicals is considered the initial step in a chain of events leading to membrane lipid peroxidation and eventually cell apoptosis or necrosis (10-13). ROS production and their leading damages would be confined by several endogenous protective mechanisms (3). When ROS formation is extreme, additional protective mechanisms of dietary antioxidants may be of great importance (14, 15).

Antioxidant nutrients and enzymes are the cooperative protective defensive systems against free radical damages (16). Antioxidant and radical scavengers have been studied on the mechanism of CCl_4_ toxicity and protect liver cells from CCl_4_ induced damage by lipid peroxidation (12). Catalase (CAT), superoxide dismutase (SOD), glutathione reductase (GR) and glutathione peroxidase (GPx) as antioxidative enzymes and glutathione (GSH), vitamins C and E as nonenzymatic antioxidants are biological antioxidants (17). Natural antioxidants such as fruits and vegetables, which provide protection against free radicals can decrease the incidence and mortality rates due to cancer and heart diseases, in addition to a number of other health benefits (18). Recently, an increasing trend of herbs usage has been seen, such as cornelian cherry (*Cornus mas*), which contains high level of antioxidants and anthocyanins. *Cornus mas* fruits have been used for gastrointestinal and excretory disorders in traditional medicine (19); also improve liver and kidney functions, and have protective effects such as antiallergic, antidiabetic, antibacterial, antimicrobial, antihistamine and antimalarial properties (20, 21).

*Cornus mas* (*C. mas*) is known as the European and Asiatic Cornelian Cherry. This plant has been used in traditional medicine for the treatment of diarrhea, intestinal inflammation, fever, malaria treatment, kidney stones and kidney and bladder infections. *C. mas* fruits contain iron, calcium, vitamins such as E, B2, B1, C, folic acid, anthocyanins, flavonoids, and plenty of oxalic acid (22, 23). It also contains antioxidant substances including butylated hydroxyanisole and butylated hydroxytoluene and has the potential in the treatment of some cancers (24, 25).

## 2. Objectives

This study was designed to investigate the protective effects of *Cornus mas* fruits extract on serum biomarkers in CCl_4_-induced hepatotoxicity in male rats.

## 3. Materials and Methods

### 3.1. Chemicals

Ethylene diamine tetra acetic acid (EDTA) and trichloroacetic acid (TCA) were obtained from Sigma-Aldrich Chemical Co. Ltd. (USA). Carbon tetrachloride (CCl_4_) and thiobarbituric acid (TBA) were obtained from Merck Co. (Germany). Assay kits for the estimation of biochemical factor such as aspartate aminotransferase (AST), alanine aminotransferase (ALT) and alkaline phosphatase (ALP) were purchased from Pars Azma (Iran).

### 3.2. Plant Material

*Cornus mas* fruits were obtained from suburbs of kaleibar (East Azarbayjan, Iran) at the end of summer 2012. The fruits were air-dried and powdered. In all steps, components were protected from direct sunlight. The powder was kept at 8˚C.

### 3.3. Extraction

Air-dried fruits of *Cornus mas* were powdered coarsely. Then 500 g of the powder was mixed with methanol: water (7:3) at 25 ± 2˚C. The solvent was completely removed by rotary vacuum evaporator at 50˚C. Then *Cornus mas* fruits extract (CMFE) was frozen at -20˚C until use.

### 3.4. Animals and Treatment

Thirty animals (Wistar strain male albino rats [250-300 g]) were kept in polypropylene cages in a room at 22 ± 2˚C with humidity of 44-55%, the light and dark cycles (12/12 h for each) for one week before and during the experiments. Animals were fed with a standard rodent pellet diet and clean drinking water ad libitum. The protocol of this study was approved by the Animal Ethic Committee of Baqiyatallah University of Medical Sciences.

Animals were divided into five groups (n = 6) as follows:

1. Group I: normal control, received drinking water orally for 14 days, on the 14th day olive oil (1 mL/kg i.p.).
2. Group II: toxic control, received drinking water for 14 days orally, on the 14th day CCl_4_ (1 mL/kg i.p.) in 1:1 dilution with olive oil.
3. Groups III and IV: treatment groups, received CMFE at the doses of 200 and 500 mg/kg orally for 14 days, respectively, on the 14th day received CCl_4_ (1 mL/kg i.p.) at the same dose, two hours after the last dose of extract administration. Group V: standard drug Silymarin, received silymarin (100 mg/kg, orally) for 14 days and on the 14th day received CCl_4_ (1 mL/kg i.p.) in 1:1 dilution with olive oil, two hours after administration of the last dose of silymarin.

### 3.5. Preparation of Serum

Animals were killed 24 hours after administration of CCl^4^. Blood samples were collected from the heart left ventricular and allowed to clot for 30 minutes. Samples were centrifuged at 2500 rpm for 15 minutes and used for biochemical analysis. The collected serum samples were frozen at -20˚C. Aspartate aminotransferase (AST), alanine aminotransferase (ALT), alkaline phosphatase (ALP) enzymes activity, total protein (TP) and albumin (Alb) in all samples were measured by standard diagnostic test kits (Pars Azma, Iran).

### 3.6. Provision of Liver Homogenate

Hepatic tissues were homogenized in KCl [10 mM] phosphate buffer (1.15%) with ethylenediamine tetra acetic acid (EDTA: pH = 7.4) and centrifuged at 3000 rpm for 30 min. The supernatant was collected to use for the measurement of malondialdehyde (MDA). Total protein content was determined based on the Lowry’s method (26).

### 3.7. Designation of Lipid Peroxidation

Liver homogenate lipid peroxidation was measured based on the formation of thiobarbituric acid reactive substance (TBARS). Two milliliters of thiobarbituric acid reagent (15% w/v TCA, 0.375% w/v TBA and 0.25 M HCl) was added to 2 mL of supernatant. The dilution was heated for 15 minutes in boiling water. After cooling, centrifuged at 1000g for ten minutes and precipitate was removed. Malondialdehyde (MDA) forms mixed with TBA, which was measured by spectrophotometer at 532 nm. The concentration of MDA was computed based on the absorbance coefficient of the TBA–MDA complex (ε = 1.56 × 10^5^/M/cm), and it was presented as nmol/mg protein (27).

### 3.8. Statistical Analysis

Data were presented as mean ± S.E. Tukey post hoc test was used to compare different parameters between the groups. The significant level was considered at P value < 0.05.

## 4. Results

### 4.1. CMFE Effect on Serum Enzymes Activity

As shown in Table 1, activities of serum ALT, AST and ALP were markedly elevated in toxic group compared to control group, indicating liver damage. Treatment with *Cornus mas* fruits extract (CMFE) in the two different dosages at 200 and 500 mg/kg for 14 days remarkably prevented CCl_4_ induced elevation of serum enzymes activity.

**Table 1.** Effect of CMFE on Serum Profile in Rat (n = 6) ^a, b^

Treatment	AST, U/L	ALT, U/L	ALP, U/L	T-Protein, g/dL	Albumin, g/dL
**Control + olive oil**	58.12 ± 5.52	43.86 ± 6.35	214.29 ± 4.83	7.85 ± 0.15	3.65 ± 0.06
**1 mL/kg CCl**_**4**_	155.74 ± 4.21^c^	197.4 ± 5.05^c^	322.63 ± 10.98 ^c^	5.83 ± 0.23 ^c^	1.31 ± 0.08 ^c^
**200 mg/kg CMFE + CCl**_**4**_	101.82 ± 4.69 ^d^	133.60 ± 5.59 ^d^	248.97 ± 6.11 ^d^	7.10 ± 0.11 ^d^	2.20 ± 0.09 ^d^
**500 mg/kg CMFE + CCl**_**4**_	115.03 ± 2.96 ^d^	116.87 ± 5.65 ^d^	235.23 ± 5.24 ^d^	7.25 ± 0.14 ^d^	2.85 ± 0.12 ^d^
**100 mg/kg Silymarin + CCl**_**4**_	85.22 ± 3.52 ^d^	72.92 ± 3.25 ^d^	223.64 ± 5.23 ^d^	7.91 ± 0.16 ^d^	3.20 ± 0.09 ^d^

^a^ Abbreviations: ALP, alkaline phosphatase; ALT, alanine aminotransferase; AST, aspartate aminotransferase; CCl_4_, carbon tetrachloride; CMFE, *Cornus mas* fruits extract.

^b^ Data are presented in Mean ± SE.

^c^ Indicates significance at P < 0.05 probability from control group.

^d^ Indicates significance at P < 0.05 probability from CCl_4_ group.

### 4.2. Effect of CMFE on Serum Level of Total Protein and Albumin

As given in Table 1, the TP and Alb concentrations in toxic group were lower compared with control group and it attained near the normal value in groups treated with CMFE.

### 4.3. Effect of CMFE on Lipid Peroxidation

**Figure 1.**
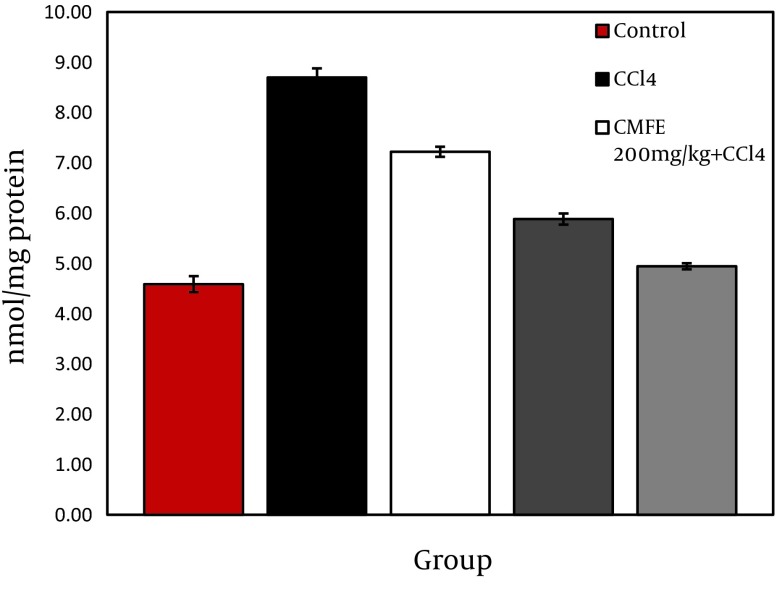
Effect of CMFE on Liver Contents of MDA in Rat * Indicates significance at P < 0.05 probability from control group; # Indicates significance at P < 0.05 probability from CCl_4_ group.

Evaluated level of Malondialdehyde content in homogenate of rat liver is shown in Figure 1. MDA content in liver homogenate was significantly (P < 0.05) increased in CCl_4_ group compared to normal control. MDA level of CMFE treatment at 200 and 500 mg/kg were significantly (P < 0.05) decreased compared to toxic (Group 2).

## 5. Discussion

Our study showed the protective effects of *Cornus mas* fruits extract (CMFE) on serum biomarkers against hepatotoxicity induced by CCl_4_ in rats. Increased generation of reactive oxygen species (ROS) has a major effect on the pathogenesis and toxicity of a wide range of compounds (28). Nowadays hepatoprotective drugs are generally used against liver damages induced by CCl_4_ (is a hepatotoxic agent). Histological signs and features of liver damage by CCl_4_ are similar to acute viral hepatitis. CCl_4_ has been commonly used in rat experimental models to investigate the oxidative stress induced in various organs. To the best of our knowledge, this is the first study to evaluate these effects of CMFE in an attempt to prevent liver damage from CCl_4_.

In liver damages, the serum level of enzymes located normally in cytosol increased due to releasing into the blood. The type and range of hepatocellular damage can be estimated by the serum level of these enzymes (29).

AST, ALT and ALP are authentic markers for liver function. Increased serum level of enzymes such as aspartate aminotransferase and alanine amino transferase have been seen in rats received CCl_4_ leading to increased cell damage, permeability, and hepatocytes necrosis. Alkaline phosphatase as a membrane bound enzyme, is excreted in association with bile when liver is affected, defective excretion increased the serum level of this enzyme (30). Treatment by oral administration of CMFE at doses of 200 and 500 mg/kg in rats decreased the mentioned enzymes activity. This event may be followed by plasma membrane stabilization and improve in CCl_4_ induced hepatic tissue damage, thus the serum levels of transaminases become normal with hepatic parenchyma healing and regeneration of hepatocytes (31). Total protein is a common laboratory test to evaluate the effect of various toxic chemicals (32). Decline in TP content can be deemed as a useful index of severity of hepatocellular damage. The lowered levels of TP and Alb recorded in the serum as well as in the liver of CCl_4_-treated rats revealed the severity of hepatopathy (33). In the present study, TP and Alb concentration were very low in rats treated with CCl_4_. In groups treated with CMFE, these markers significantly (P < 0.05) increased compared to the toxic group and the values were closer to those of the control group.

Oxidative stress initiates lipid peroxidation of cell membranes polyunsaturated fatty acids (34). Lipid peroxidation represents one of the most frequent reactions resulting from free radicals invasion on biological structures and by cumulating oxidized lipids in the cell membrane (35). Our result showed the reduction effect of CMFE on TBARS production. In this study, CCl4 administration significantly (P < 0.05) increased the hepatic MDA content, which may indicate an increase in lipid peroxidation. Significant decrease in the hepatic malondialdehyde content, as a marker of lipid peroxidation, confirmed that treatment with CMFE could have a great protective effect against the CCl_4_-induced hepatic lipid peroxidation. This protection mechanism preserves the liver from toxin induced damages through the hepatic regeneration stimulating and liver lipid peroxidation inhibiting (36).

*Cornus mas* fruit extract effect may be due to some antioxidant components, leading to membrane stabilizing and normalization of fluctuated biochemical profiles induced by CCl_4_ exposure. Therefore, plant extract compounds effect on the liver is to keep its normal function and decrease derangements of cell membrane. Purification of the active component(s) of *Cornus mas* to determine the exact protective effects on hepatocytes is recommended for further studies.
